# Influence of Childhood Family Routines on Adult Depression: A Cross Sectional Study

**DOI:** 10.3389/fpsyg.2021.654433

**Published:** 2021-07-05

**Authors:** Yuzhi Yan, Junyi Zhang, Shenghong Dong

**Affiliations:** ^1^School of Psychology, Jiangxi Normal University, Nanchang, China; ^2^Department of Psychology, School of Social and Behavioral Sciences, Nanjing University, Nanjing, China

**Keywords:** family routines, tolerance of uncertainty, rumination, depression, multiple mediating effect

## Abstract

In order to explore the influence of childhood family routines on adult depression and the mediating role of tolerance of uncertainty and rumination, the current study tested 818 participants by adopting four questionnaires. The results of structural equation modeling revealed that (1) family routines had a negative effect on depression among Chinese college students; (2) family routines were found to have a positive effect on tolerance of uncertainty, and tolerance of uncertainty was a bridge linking family routines and depression; (3) family routines had a significant effect on depression through rumination; (4) the relationship between family routines and depression was partially mediated by the chain of tolerance of uncertainty and rumination. The result reveals not only the fact that childhood family routines have significant influence on college students' depression but also the mechanism of childhood family routines that affect college students' depression. The limitations and implications of our study were also discussed.

## Introduction

Depression has an adverse effect on individual's work performance, interpersonal relationships, economic status, and even suicidal inclination (Muharam et al., 2017). It has been reported that the victims have reached more than 322 million worldwide (World Health Organization, 2017). Although depression is a mental illness, it also affects physical health, increases morbidity, and mortality (Brown et al., 2011). Depression is ranked by the WHO as the single largest contributor to global disability (In 2015, 7.5% of population were reported to live with disability). Depression is also the major contributor to suicidal death, which numbers around 800,000 per year (World Health Organization, 2017). Unlike many physical diseases, the worse the condition of patients with depression, the lower their willingness to receive treatment (Sawyer et al., 2012). Therefore, it is very important to carry out etiological research for effective prevention and intervention of depression.

Many studies have shown that the family environment plays an important role in the occurrence, maintenance and development of depression (Ivanova and Israel, 2005; Manczak et al., 2016). However, previous research of family routines shows that few studies have focused on the influence of family environment on depression. Compared with other treatment methods that require a lot of professional knowledge and professional training, the treatment of depression from the perspective of family routines, which are integrated into the patient's family daily life, has the characteristics of safety, low cost-effectiveness, easy acceptance, and easy promotion (Manczak et al., 2016). Therefore, it is necessary to conduct more in-depth study of the influence of family routines on depression and its mechanism.

### Family Routines and Depression

In family life, daily activities such as sleeping, eating, housework and leisure are often involved. Are these activities orderly or disorderly? Family routines is a variable used to describe the characteristics of this family environment. It refers to the behaviors that occur repeatedly in family life. These behaviors occur repeatedly at the same time or place which are directly performed by young people. They are regular, observable and easy to predict (Boyce et al., 1983; Manczak et al., 2016). Typical sleeping routines might include bathing, dressing, brushing teeth, saying goodnight to family members, reading stories, and lying in the bed. Family routines is a fixed pattern adopted by family members in daily activities, and it is a predictable, stable and structured behavior unit in the family (Denham, 2002). In a family that has established good family routines, daily activities are arranged in an orderly and regular manner, and thus children would always know how to do as well as when to do them. Otherwise, daily activities are chaotic, unpredictable, and disorderly. Since the concept of family routines was proposed, it has aroused widespread interest from foreign researchers and conducted a large number of studies (Sytsma et al., 2001; Kitsaras et al., 2018). A combination of these studies shows that predictable, stable and structured family routines are conducive to children's sense of security, trust, independence (Sytsma et al., 2001), autonomy (Zhang, 2010), self-emotion regulation (Fleer et al., 2017), sense of control (Pruitt, 1998), and other positive psychological characteristics which serve as protective factors for children's physical and mental development. At the same time, a good level of family routines has a significant effect on enhancing children's adaptability (Mcloyd et al., 2008) and reducing internalization and externalization behavior problems (Harris et al., 2014). Symptoms of anxiety and depression often occur concurrently or sequentially in adolescents (Garber and Weersing, 2010). Previous studies showed 25% to 50% of adolescents with a primary depression also had anxiety, while 10–15% of adolescent who met criteria for anxiety suffered from depression (Garber and Weersing, 2010; Cummings et al., 2014). A study in 2020 conducted a cross-sectional study among Chinese students and found the prevalence of a combination of depressive and anxiety symptoms was 31.3% (Zhou et al., 2020). However, by combing previous studies, we can see that existing studies have mainly focused on exploring the influence of family routines on the physical and mental development of childhood (Lanza and Drabick, 2011; Kitsaras et al., 2018), while research on the influence of individual's physical and mental development in adulthood is relatively lacking. Some studies have suggested that the influence of childhood family routines on childhood depression may also affect depression in adulthood (Manczak et al., 2016), which needs more support from empirical research. Therefore, in order to further promote this field, the current study takes depression as the starting point, adopts retrospective examination, and takes adult college students as subjects to explore the influence of childhood family routines on individual's physical and mental development and to verify its mechanism. As children's sense of predictability and control formed by regular activities or events in the family routines are protective factors to prevent depression (Ivanova and Israel, 2005), the first research hypothesis is: There will be a negative relationship between childhood family routines and depression of adult college students. The higher the level of childhood family routines, the lower the risk of depressive symptoms in adulthood.

### Family Routines, Tolerance of Uncertainty, and Depression

Tolerance of Uncertainty (TU) refers to individual's differences in cognitive, emotional, or behavioral response propensity when responding to uncertain situations (Zvolensky et al., 2010; Li et al., 2015). Becker and Knudsen (2005) found that decision makers would better be able to cope with pervasive uncertainty if their strategic responses were constrained by routinized behavior. There are also many studies showing that tolerance of uncertainty is significantly related to negative emotions such as anxiety and depression (Buhr and Dugas, 2006; Cai et al., 2018; Rettie and Daniels, 2020). In the face of uncertainty, individuals with low tolerance of uncertainty are easily stressed and distressed. They believe that uncertainty is negative and should be avoided, which often leads to functional effects (e.g., overwhelmed, delayed decision-making). However, the world is full of uncertainty, so they are more likely to suffer from anxiety and depression in their lives (Buhr and Dugas, 2006).

Some studies have explored the mediating role of tolerance of uncertainty in the influence of family factors on individual's emotions. For example, Zlomke and Young (2009) chose college students as subjects and found that tolerance of uncertainty was a mediator between perceived anxious rearing and both worry and anxiety. Wu (2016) adopted adolescents as subjects and found that tolerance of uncertainty plays a mediating role between parental psychological control and depression. So, does tolerance of uncertainty play a mediating role between family routines and depression? Studies have found that family routines affect adolescents' emotional regulation and self-control abilities. The higher the level of family routines, the stronger the emotional regulation (Miller et al., 2016) and self-control (Pruitt, 1998). Research also shows that the individual's sense of control affects the level of tolerance of uncertainty (Buhr and Dugas, 2006). The stronger the individual's sense of control, the higher the level of tolerance of uncertainty. Cai et al. (2018) took 14~24 years old with autism spectrum disorder as subjects and found that emotion regulation affects anxiety and depression through tolerance of uncertainty. This illustrates that family routines may affect the individual's tolerance of uncertainty level by affecting the individual's self-control ability and emotional regulation ability, thereby modulating their depression level. Based on this, the second research hypothesis is: Childhood family routines affect tolerance of uncertainty, which in turn affects college students' depression.

### Family Routines, Rumination, and Depression

Rumination refers to the repeated thinking and behavior of individuals about the symptoms, causes and consequences of past painful experiences (Nolen-Hoeksema, 1991, 2000). The RESPONSE style theory proposed by Nolen-Hoeksema (1991) believes that rumination is a maladaptive RESPONSE style, which may intensify and prolong individual negative emotions by increasing negative thoughts, hindering problem-solving skills and social interaction (Nolen-Hoeksema, 2000; Nolen-Hoeksema et al., 2008). Many studies have confirmed that rumination can cause depression (Olatunji et al., 2013; Wang et al., 2018). For example, a meta-analysis has found that high levels of rumination can predict high levels of depression (Olatunji et al., 2013).

A large number of studies have shown that rumination plays a mediating role in the influence of some depression risk factors on individual depression (Kovács et al., 2021). For example, research by Zeng et al. (2015) found that rumination plays an intermediary role between childhood psychological abuse and college students' trait depression. So, does rumination play a mediating role between family routines and depression? Studies have shown that individual rumination is affected by emotional regulation. For example, studies have revealed that emotional regulation ability is significantly negatively correlated with trauma-related rumination (Ehring and Ehlers, 2014). Some researchers even believe that rumination is a dysfunctional emotion regulation strategy (Nolen-Hoeksema et al., 2008). Studies have shown that self-control ability affects the level of rumination. When rumination occurs, self-control can help the individuals to break away from rumination and effectively reduce the level of rumination (Denson et al., 2011). Ample researches have shown that family routines can affect adolescents' emotional regulation and self-control. The higher the level of family routines, the stronger the emotional regulation (Miller et al., 2016) and self-control (Pruitt, 1998). This indicates that family routines may affect individual's self-control and emotional regulation abilities, thereby affecting their rumination and thus their depression levels. Therefore, the third research hypothesis is: Childhood family routines affect rumination, and moreover rumination affects college students' depression.

### Family Routines, Tolerance of Uncertainty, Rumination, and Depression

Liao and Wei (2011) chose college students as subjects and found that rumination plays a mediating role between tolerance of uncertainty and depression. Yook et al. (2010) have similar findings in patients with major depression. The reason may be that individuals with low tolerance of uncertainty are intended to adopt repetitive thinking (such as worrying, rumination) to cope with stressful situations (Buhr and Dugas, 2006). On this basis, the fourth research hypothesis is: Tolerance of uncertainty and rumination play a chain of intermediary role in the association between family routines and college students' depression.

**Figure 1:**
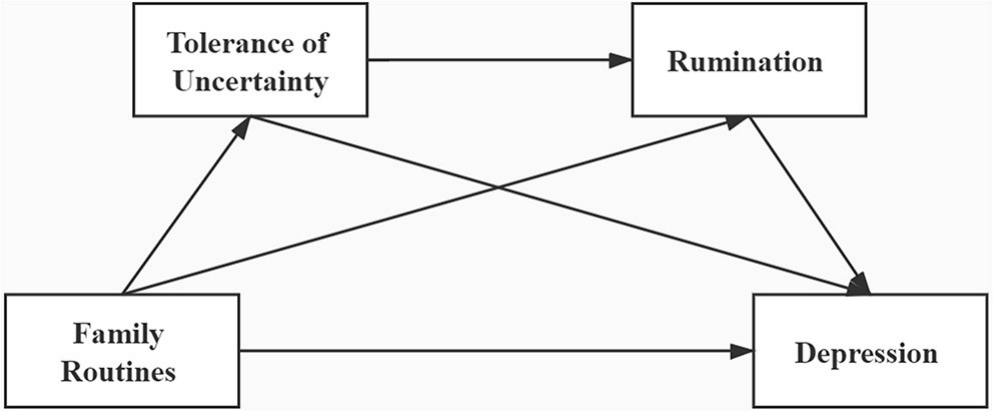
Hypothesized model.

In summary, this study has two research purposes: 1. To examine whether family routines during childhood would be significantly correlated to depression in adult college students. 2. Examine the individual and chain mediating effects of tolerance of uncertainty and rumination between tolerance of uncertainty and college students' depression (as shown in Figure 1). According to Hayley et al. (2015), and early-adulthood is a critical period for individual physiological and psychological development, while college students are generally in the age stage of 18–25 years old, which is an important part of the early-adulthood group (Chen et al., 2021). In China, the prevalence of depression among college students has been increasing in recent 10 years (Wang et al., 2020), so it is important to probe into the sample situation of Chinese college students. Hence, we chose college students as subjects and adopted retrospective examination methods (Zlomke and Young, 2009) in this study, it could be more accurately to deepen our understanding of the relationship between post-adult depression and childhood family routines, and answer the question “how” the childhood family routines affect post-adult depression, as well as revealing the “black box” between the two, in order to provide a theoretical basis for the prevention and intervention of depression among college students.

## Methods

### Participants and Procedure

Data collection was conducted by the methods of cluster random sampling, and participants were recruited through three universities in Jiangxi province. This study was approved by the Ethics Committee of the School of Psychology of Jiangxi Normal University, and all participants signed the written informed consent. Students of psychology major distributed questionnaires in class. In the part of guidance, the authenticity of the answer and the confidentiality principle of personal information were emphasized. In total, 889 students participated in the study, and 818 valid questionnaires were obtained by excluding extreme data and unfinished test data (male=155, female = 663; age: M = 20.21, SD = 4.94), with an effective rate of 92.01%.

### Measures

#### Family Routines Scale

The Family Routines Scale (FRS) was developed by Zhang (2010), who adopted Jensen et al.'s (1983) family routines scale and combined it with the context of actual family situation in China. It was used for retrospective examination of childhood family routines. The scale includes 21 items, using a 5-point scale of “1” for “Completely consistent” and “5” for “Completely inconsistent.” A lower score indicates a higher degree of family routines level. The family routines scale has a good reliability, and its α coefficient is 0.860. In this study, the α coefficient is 0.915, the validity index of the model fits well [*x*^2^/*df* = 6.51(*p* < 0.001), CFI = 0.94, NNFI = 0.93, RMSEA = 0.09, SRMR = 0.068].

#### Intolerance of Uncertainty Scale

The Intolerance of Uncertainty Scale (IUS) is a Chinese version of the IUS scale revised by Li et al. (2015) based on Freeston et al. (1994). The scale contains 11 items in total. It uses five points, from 1 (Completely inconsistent) to 5 (Completely consistent). A lower score indicates a higher level of tolerance of uncertainty. The revised IUS has a good reliability and the α coefficient is 0.888 in this study. The validity index of the model fits well [*x*^2^*/df* = 6.74(*p* < 0.001), CFI = 0.97, NNFI = 0.96, RMSEA = 0.086, SRMR = 0.043].

#### Ruminative Responses Scale

The Ruminative Response Scale (RRS) was developed by Nolen-Hoeksema (1991) and revised by Han and Yang (2009). Participants responded to 22 items on a 4-point Likert-type scale from 1 (Never) to 4 (Always), a higher score indicating a higher level of rumination. The scale contains three dimensions: Symptom rumination, Brooding, and Reflective pondering. The revised RRS has a good reliability and the α coefficient is 0.920 in this study, and the validity index of the model fits well [*x*^2^/*df* = 5.11(*p* < 0.001), CFI = 0.96, NNFI = 0.95, RMSEA = 0.076, SRMR = 0.053].

#### Self-Rating Depression Scale

The Self-rating Depression Scale (SDS) was developed by Zung (1965) and revised by Wang et al. (1999). Participants responded to 20 items on a 4-point Likert-type scale from 1 (Never or Occasionally) to 4 (Always), and a higher score indicates a higher level of depression. The revised SDS has a good reliability and the α coefficient is 0.641 in this study. The validity index of the model fits well [*x*^2^/*df* = 3.52(*p* < 0.001), CFI = 0.91, NNFI = 0.90, RMSEA = 0.059, SRMR = 0.052].

## Results

### Common Method Deviation Test

Common method bias may exist because all of the data were generated by self-report, which may decrease the validity of the results. Generally, there are two ways of controlling common method bias, procedural remedies and statistical remedies. Procedural remedies refer to control measures incorporated into the process of a study's design and measurement by researchers. In this study, we kept strict principles of confidentiality and voluntarism, and asked participants to truthfully fill out each item in the questionnaire. We used the class collective measured approach to acquire the data, and recycled the questionnaires immediately after each survey was completed. The directions of all questionnaires were unified in the same format. These can partially control the Common method bias.

In addition, statistical remedies involve a statistical test that is applied after data collection. A confirmatory factor analysis (CFA) was used to test the common method bias in this study, and the one factor model fitting indexes were χ^2^= 15888.98, *df* = 2627, χ^2^/*df* = 6.05, RMSEA = 0.12, CFI = 0.58, NFI = 0.56, NNFI = 0.57, and SRMR = 0.11, which indicates that there was no serious common method bias problem in each variable in this study.

### Descriptive Statistics

The mean value, standard deviation, and correlation coefficients of each variable were statistically analyzed using SPSS 23.0. As shown in Table 1, the mean value and standard deviation of each variable were within the acceptable range. Family routines are significantly positively correlated with tolerance of uncertainty, while negatively correlated with rumination and depression. Tolerance of uncertainty is significantly negatively correlated with rumination and depression, and rumination is positively correlated with depression. The results of descriptive statistics and related analysis preliminarily illustrate the relationship between variables, providing a basis for further data analysis.

**Table 1:** Means, standard deviations, and correlations among study variables.

**Variables**	***M* (*SD*)**	**1**	**2**	**3**	**4**
1. Family Routines	62.292 (13.774)	1			
2. Tolerance of Uncertainty	35.163 (7.600)	0.120^**^	1		
3. Rumination	45.830 (10.613)	−0.160^**^	−0.443^**^	1	
4. Depression	41.770 (7.060)	−0.135^**^	−0.284^**^	0.386^**^	1

*N = 818;*

** *p < 0.01*.

### Structural Equation Model Analyses

LISREL 8.80 and the SPSS Macro Program PROCESS (version 3.3) were used to test the structural equation model. The results are represented in Tables 2, 3. Firstly, the main effect was tested, with family routines as the independent variable and depression as the dependent variable to construct the structural equation model 1. The results show that family routines significantly affect depression (β = −0.146, *p* < 0.001), and H1 is supported.

**Table 2:** Regression analysis of the chain mediation model.

**Regression equation**		**Overall model fit**			**Significance of regression coefficient**	
**Outcome**	**Predictor**	***R***	***R^**2**^***	***F***	**β**	***t***
TU	FR	0.126	0.016	13.153	0.126	3.627^***^
R	FR	0.438	0.192	96.939	−0.090	−2.822^**^
	TU				−0.418	−13.171^***^
D	FR	0.375	0.141	44.459	−0.090	−2.742^**^
	TU				−4.457	−4.456^***^
	R				0.250	6.904^***^

*FR, Family Routines; TU, Tolerance of Uncertainty; R, Rumination; D, Depression*.

** *p < 0.01*,

*** *p < 0.001*.

**Table 3:** Results of specific mediation effect based on bootstrapping.

**Effect**	**Intermediate path**	**Effect value**	**Boot standard error**	**95% confidence interval**	
				**Lower limit**	**Upper limit**
Direct effect	FR → D	−0.050	0.018	−0.0854	−0.0141
Intermediary effect	FR → TU → D	−0.031	0.008	−0.0482	−0.0151
	FR → R → D	−0.011	0.005	−0.0209	−0.0034
	FR → TU → R → D	−0.012	0.006	−0.0245	−0.0015

*FR, Family Routines; TU, Tolerance of Uncertainty; R, Rumination; D, Depression*.

Secondly, models 2 and 3 were established with tolerance of uncertainty and rumination as single mediators. Through the model 4 of the SPSS Macro Program PROCESS, the bootstrap method was used to repeat the sampling 5,000 times to test the mediating effect. The results show that the mediating effect of tolerance of uncertainty was −0.0184, with 95% confidence interval [−0.0309, −0.0068], excluding 0, thus verifying H2. The mediating effect of rumination was −0.0249, with 95% confidence interval [−0.0412, −0.0103], excluding 0. Based on the results, H3 was verified.

Finally, the chain multiple mediation effect was tested. According to the model 6 of the SPSS Macro Program PROCESS, the 95% confidence interval of the mediating effect was estimated by extracting 5,000 bootstrap samples, and the chain multiple mediation effect of tolerance of uncertainty and rumination was tested significantly. The results are shown in Table 3. Family routines → tolerance of uncertainty → depression mediating effect is −0.031. 95% confidence interval is [−0.0482, −0.0151], excluding 0, which shows mediating effect is significant. The mediating effect of Family routines → rumination → depression is −0.011, and the 95% confidence interval is [−0.0209, −0.0034], excluding 0, suggesting the mediating effect is significant. Chain multiple mediating effect of Family routines → tolerance of uncertainty → rumination → depression is −0.012, with 95% confidence interval [−0.0145, −0.0015], excluding 0, which indicates that H4 is verified.

**Figure 2:**
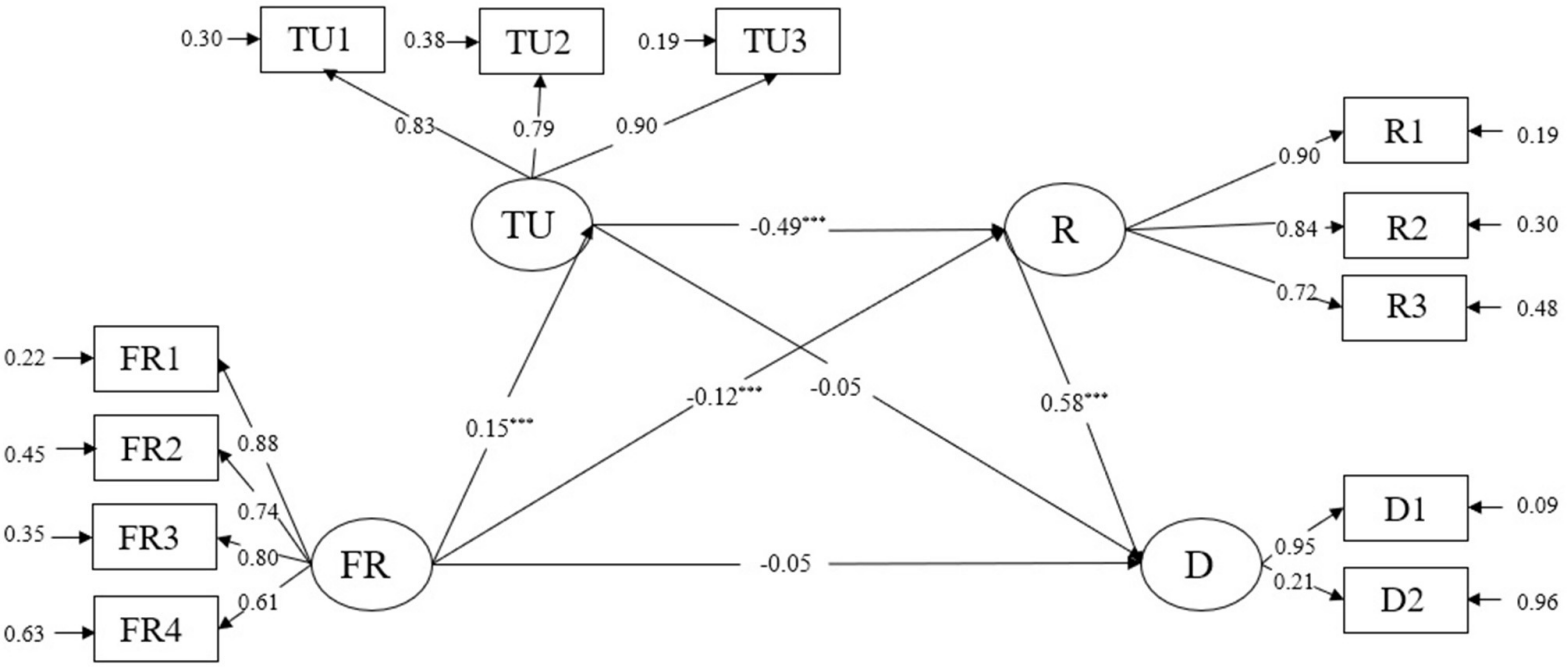
The standardized path coefficients in model testing. FR, Family Routines; TU, Tolerance of Uncertainty; R, Rumination; D, Depression. ***p < 0.001.

The results of structural equation model analysis using LISREL 8.80 show that the index of the model fits well (*x*^2^/*df* = 5.141(*p* < 0.001), CFI = 0.96, NFI = 0.96, NNFI = 0.95, RMSEA = 0.074, SRMR = 0.042], indicating that the model can be well-established. According to the above analysis, the chain mediation model can be constructed (see Figure 2).

## Discussion

### The Influence of Childhood Family Routines on Depression of Adult

Conventional family studies have mostly focused on the impact on the physical and mental development of childhood (Lanza and Drabick, 2011; Kitsaras et al., 2018). However, little research has been conducted on the physical and mental development of adulthood. This study uses retrospective examination to explore the influence of childhood family routines on adult college students' depression and its mechanism. The results have verified our research hypotheses, deepened the understandings of family routines studies, and expanded the study on the etiology of depression. The results of the study show that family routines demonstrate significant relationships to depression, that is, the higher the level of family routines in childhood, the lower the risk of depressive symptoms in adulthood.

Why do family routines in childhood still have an impact on depression in adult college students after their childhood? On the one hand, the reason might be that the unpredictability of unstable and unstructured family routines for family activities increases the risk of depression in adulthood. There are ample studies proposing that an individual's unpredictable experience is closely related to depression-related processing such as learned helplessness, perception of stress, psychological well-being, and physiological stress response (Manczak et al., 2016). On the other hand, this could be related to children's sense of security, trust, independence (Sytsma et al., 2001), autonomy (Zhang, 2010), and sense of control (Pruitt, 1998) in a stable and structured family routines. It is related to positive psychological traits such as good self-regulation ability (Fleer et al., 2017). Once these psychological traits are formed, they become protective factors to prevent depression, serving as the early “vaccination” for the depression when they enter adulthood. For example, a recent study showed that emotional suppression strategies play a mediating role between psychological neglect and depression (O'Mahen et al., 2015). Autonomy, as a potential personality feature, is closely related to depression, the higher an individual's level of autonomy, the more conducive to alleviating their depressive symptoms (Bieling and Alden, 2001).

### Construction of the Chain Mediation Model

The results of this study found that family routines significantly correlated to individual tolerance of uncertainty, and affect depression through tolerance of uncertainty. It demonstrates that children would be in a chaotic, unpredictable and uncertain environment and thus result in a lack of sense of control over the environment if good and structured family routines are not established in the family. Once a child develops low tolerance of uncertainty, low tolerance of uncertainty will force him be more inclined to feel anxiety and depression when he encounters uncertain situations or events, even after his adulthood (Buhr and Dugas, 2006; Cai et al., 2018). In addition, although there are endless research on tolerance of uncertainty, there are few studies taking the reasons for the differences in the tolerance of uncertainty levels of different individuals into account. Zlomke and Young (2009) found that the anxious parenting style of parents easily leads to the formation of low tolerance of uncertainty. This research shows that the predictable, stable, and structured level of family activities in the family environment will also affect the individual's tolerance of uncertainty.

The results of this study also show that family routines significantly correlated to individual rumination, and affect depression through rumination. Some researchers have pointed out that the reason for rumination is linked to early childhood experience (Nolen-Hoeksema et al., 1995). The results of this study support this theoretical hypothesis. Combining with the previous research, this study reveals the influence of family routines on tolerance of uncertainty and rumination, further emphasizing the key role of family routines in individual self-development. Why do family routines influence depression through rumination? The underlying reason might be that predictable, stable and structured family routines are conducive to the formation of good self-control and emotional regulation abilities in children. Once rumination appears, these abilities help individuals to break away from rumination (Denson et al., 2011), thereby reducing the level of rumination and reducing depression. Of course, this is just a theoretical speculation, and related research needs to be further conducted.

Finally, this study also found that family routines can also affect depression through the chain-mediated role of tolerance of uncertainty and rumination. This shows that individuals with low tolerance for uncertainty may think repeatedly, hoping to learn more about the situation, and thereby reducing their sense of uncertainty (Liao and Wei, 2011). However, the use of rumination with low tolerance of uncertainty to cope with and manage stress may lead to worse result and even alleviate stress (Nolen-Hoeksema, 1991), which in turn is associated with greater depression and anxiety. The chain mediation effect reveals how family routines affect depression through both separate and joint effects of the two mediating variables, tolerance of uncertainty and rumination, elaborating the affecting mechanism of family routines on depression. It has important theoretical and practical significance in the intervention and prevention of depression among college students.

### Limitations and Future Research Directions

The current research inspires us to pay attention to the influence of family routines on individual development, because it may last a lifetime. In order to provide a good family environment for children's development, it is of great importance to establish predictable, stable, and structured family routines, and to decrease the occurrence of depression by improving individual tolerance of uncertainty and reducing the level of rumination. This study still has the following limitations and deficiencies. First, in terms of research methods, the cross-sectional data collection makes it hard to make causal attributions and result application of the results to other populations. To address this limitation in subsequent research, prospective multi-site studies should be undertaken to allow the results to be generalized to a larger population of individuals. Second, participants provided retrospective reports of their childhood family routines. Although many studies in this area have adopted such research methods (Ballash et al., 2006; Zlomke and Young, 2009), it still represents less than ideal circumstances. Future research should exclude the possibility of some subjects with severe depression who reported low-level family routines due to retrospective reporting bias. For example, a combination of parental evaluation and retrospective examination should be adopted. Third, this study chose college students as subjects because early-adulthood is a critical period for individual physiological and psychological development (Hayley et al., 2015), and college students are generally in the age stage of 18–25 years old, which is an important part of the early-adulthood group (Chen et al., 2021). In China, the prevalence of depression among college students has been increasing in recent 10 years (Wang et al., 2020), so it is important to probe into the sample situation of Chinese college students. But further studies should also take a wider sample of adults into consideration. Finally, this study explored the impact of family routines on depression from the perspective of children. In fact, family routines also have an impact on parents' mind and body (Sytsma et al., 2001). Therefore, we could conduct some research to explore the influence of family routines on parents' depression in the future.

## Data Availability Statement

The raw data supporting the conclusions of this article will be made available by the authors, without undue reservation.

## Author Contributions

YZY and JYZ conducted the statistical analyses and wrote and revised the manuscript, as well as the management of the original data. SHD conceptualized the aims and hypotheses, wrote and revised the manuscript, participated in the overall design and coordination of the original data collection, performed the original data measurements, and helped perform the statistical analysis in the current study. All authors gave critical comments, read, and approved the final manuscript.

## Conflict of Interest

The authors declare that the research was conducted in the absence of any commercial or financial relationships that could be construed as a potential conflict of interest.
