# Supplementary material for: Influence of Childhood Family Routines on Adult Depression: A Cross Sectional Study
Source: Front Psychol. 2021 Jul 5;12:654433. doi: 10.3389/fpsyg.2021.654433 (PMC8288247; doi:10.3389/fpsyg.2021.654433)
Supplement: Supplementary file 1 [file Data_Sheet_1.docx]

**Appendix 1. *Family Routines Scale***

Please be assured that the answers are neither right nor wrong and will not affect you and tick the box that matches your real opinion.

| 1 | Whenever someone at home has a birthday, we remind each other.  (1 Completely consistent → 5 Completely inconsistent) |
| --- | --- |
| 2 | Most things in the family will be discussed with the whole family.  (1 Completely consistent → 5 Completely inconsistent) |
| 3 | Family members often associate with relatives.  (1 Completely consistent → 5 Completely inconsistent) |
| 4 | … |

**Appendix 2. *Intolerance of Uncertainty Scale***

The questionnaire mainly describes people's reactions to uncertain situations in life. Please read the descriptions of the following questions carefully and choose the answer that suits you best.

| 1 | If I don't know what will happen tomorrow, I can't relax.  (1 Completely consistent → 5 Completely inconsistent) |
| --- | --- |
| 2 | When I think things are uncertain, I can't go on.  (1 Completely consistent → 5 Completely inconsistent) |
| 3 | I must get rid of the uncertainty.  (1 Completely consistent → 5 Completely inconsistent) |
| 4 | … |

**Appendix 3. *Ruminative Responses Scale***

Everyone will experience some depressed things more or less, and then they will think or do something different. Please imagine what you think or do when you are depressed (not what you should do), “Never” is 1, “Sometimes” is 2, “Often” is 3, “Always” is 4.

| 1 | I often think how lonely I am. (1 never → 4 Always) |
| --- | --- |
| 2 | I often think, "If I can't stop thinking about this, then I can't continue to do what I'm doing.".(1 never → 4 Always) |
| 3 | I often think about my feeling of fatigue and pain. (1 never → 4 Always) |
| 4 | … |

**Appendix 4. *Self-rating Depression Scale***

Please tick the box that matches you according to your actual situation in the last week.

| 1 | I feel depressed. (1 never → 4 Always) |
| --- | --- |
| 2 | I think the morning of the day is the best. (1 never → 4 Always) |
| 3 | I burst into tears or want to cry. (1 never → 4 Always) |
| 4 | … |
